# Silver-loaded nanoemulsion of *Nepeta glomerulosa* extract enhances cytotoxicity and induces apoptosis in A549 and AGS cancer cells

**DOI:** 10.1186/s43046-026-00373-8

**Published:** 2026-07-06

**Authors:** Sepideh Sahebi, Golaleh Mostafavi, Seyyedeh Mahdokht Maddah

**Affiliations:** Department of Biology, YI.C., Islamic Azad University, Tehran, Iran

**Keywords:** Gastric cancer, Gene expression, Lamiaceae, Lung cancer, Nanoemulsion, *Nepeta glomerulosa* extract

## Abstract

The global rise in cancer occurrence, together with the spread of multidrug resistance, highlights the critical demand for innovative treatment strategies. Nano-delivery approaches have emerged as promising solutions that enhance bioavailability and facilitate targeted drug delivery. This study evaluated the anticancer efficacy of *Nepeta glomerulosa* extract and its silver-loaded nanoemulsion (Ag-N.g.Ext. NEs) against human lung (A549) and gastric (AGS) cells.

**Methods**

*Nepeta glomerulosa* was collected from the Binaloud Mountains, and a hydroalcoholic extract was obtained by maceration. Ag-N.g.Ext. NEs were prepared via high-energy emulsification and characterized by FTIR, SEM, DLS, ZP, and XRD. Cytotoxicity and apoptosis were assessed using MTT assay, DAPI staining, and Real-time PCR of TP53 and BAX genes.

**Results**

Ag-N.g.Ext. NEs were spherical (confirmed by SEM) with a PSA (DLS) size of 269.5 nm, a negative zeta potential of -23.1 mV, and a non-crystalline structure. The IC₅₀ of the extract for A549 and AGS was 500 and 250 µg/mL, while the Ag-N.g.Ext. NEs showed enhanced potency with an IC₅₀ of 125 µg/mL. Apoptosis and gene expression were significantly higher in Ag-N.g.Ext. NEs-treated cells (*p* ≤ 0.05). TP53 expression increased 3.317-fold in A549 and 8.027-fold in AGS; BAX increased 3.877-fold in A549 and 2.351-fold in AGS.

**Conclusions**

Nanoemulsion-based delivery significantly enhances the anticancer efficacy of the extract by promoting apoptosis, offering a promising strategy for plant-derived therapeutics.

**Graphical Abstract:**

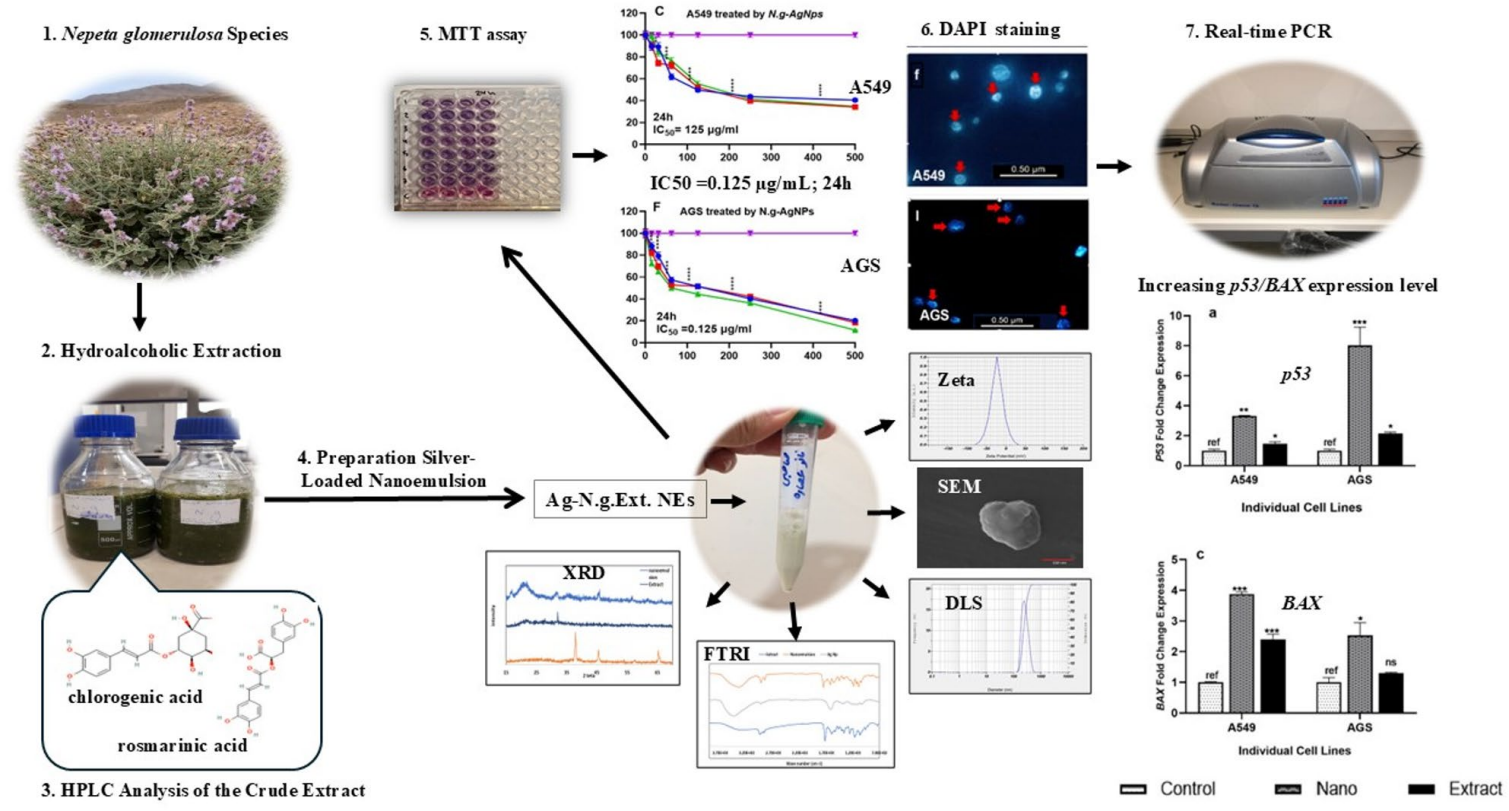

## Introduction

Cancer poses a growing global burden, with over 20 million new cases projected annually by 2025 [1]. Lung cancer remains one of the most prevalent and fatal cancers, with approximately 2 million new cases and 1.8 million deaths per year [2]. Meanwhile, gastric cancer is ranked as the fifth most common malignancy and the fourth leading cause of cancer-related deaths, responsible for over 1.08 million cases and 770,000 deaths annually [3]. These alarming statistics underscore the urgent need to develop more effective and less toxic therapeutic strategies, such as conventional chemotherapy and chemoradiotherapy, which often result in limited efficacy and severe adverse effects [2, 3].

Due to the limitations of conventional treatments, there has been increasing interest in therapeutic agents derived from natural sources, particularly medicinal plants [4]. Several phytochemicals, including sulforaphane, flavonoids, and phenolic compounds, have demonstrated potent anticancer activity in preclinical studies, and some have progressed to clinical applications. Classic examples include vinca alkaloids and paclitaxel, both of which are widely used as frontline chemotherapeutic agents. Nevertheless, many plant-derived compounds still face critical challenges, including low aqueous solubility, limited bioavailability, poor tumor selectivity, and suboptimal efficacy. These limitations highlight the need for advanced delivery systems that can enhance pharmacological performance and tumor targeting [4, 5].

Nanotechnology-based delivery platforms, particularly nanoemulsions, have emerged as highly promising carriers for anticancer therapeutics. Nanoemulsions are kinetically stable colloidal systems with droplet sizes typically in the nanometer range. While they are frequently formulated as oil-in-water (O/W) systems to enhance the delivery of hydrophobic drugs, they can also exist as water-in-oil (W/O) or even multiple nanoemulsions (e.g., W/O/W or O/W/O), depending on the surfactant properties and the intended application [6–9]. These versatile systems offer multiple advantages, including improved solubility of poorly water-soluble bioactive compounds, protection against enzymatic degradation, enhanced cellular uptake, and controlled drug release. Furthermore, their biocompatibility, along with the use of ‘generally recognized as safe’ (GRAS) excipients, ensure stability and safety, supporting their potential clinical translation [6, 7, 10, 11].

The genus *Nepeta* L. (Lamiaceae), comprising approximately 300 species distributed across Eurasia, is well known in ethnomedicine for its diverse pharmacological properties [12, 13]. *Nepeta glomerulosa* Boiss. is a semi-endemic species native to mountainous regions of Iran, including Khorassan–Kopet Dagh, and the Zagros and Alborz Mountain ranges [14]. Phytochemical studies of *N. glomerulosa* reveal diverse bioactive metabolites, notably nepetalactones, monoterpenes (e.g., 1,8‑cineole, linalool), and sesquiterpenes. Traditionally, this species has been used for its anti-rheumatic, antiseptic, wound-healing, and carminative properties [13, 15, 16]. Recent investigations highlight the anticancer potential of the aqueous extract from wild-grown *N. nuda* leaves, primarily due to their cytotoxicity against MDA-MB-231 and MCF7 breast cancer cells, HT29 and Colon 26 colon cancer cells, and HepG2 liver cancer cells [17]. Additionally, the ethanol extract of *Nepeta deflersiana* Schweinf. Ex-Hedge has been reported to exhibit anticancer activity against the lung cancer cell lines NCI-H460 and A549 [18].

Based on these findings, the present study aimed to develop a silver-loaded nanoemulsion incorporating *N. glomerulosa* extract and to evaluate its anticancer potential against human lung (A549) and gastric (AGS) adenocarcinoma cell lines, selected as representative epithelial carcinoma models from two high-burden cancers. The study further examined apoptosis induction, changes in cancer-related gene expression, dose-response effects, and preferential cytotoxicity toward cancer versus normal cells under the tested conditions. To the best of our knowledge, the anticancer activity of *N. glomerulosa* extract and its silver-loaded nanoemulsion has not previously been evaluated in A549 and AGS cells. Moreover, *Nepeta* spp. are aromatic herbs used traditionally for stomach and respiratory disorders and explored for food applications, supporting the relevance of gastric and lung epithelial models [12].

## Materials and methods

### Materials and reagents

Pre-formed stabilized silver nanoparticles (AgNPs, 20–50 nm) were obtained from Nanonyx (Tehran, Iran). According to the manufacturer, these AgNPs were prepared by chemical reduction, exhibiting a spherical morphology with a TEM-confirmed size of 20–50 nm and a negative surface charge to ensure long-term stability.

Medium Chain Triglycerides (MCT) oil fortified with Vitamin D3 was purchased from DSM (Heerlen, Netherlands), and Tween 80 (polysorbate 80) was sourced from Sigma-Aldrich (St. Louis, MO, USA). For HPLC analysis, analytical-reagent grade ethanol and methanol were purchased from Merck (Darmstadt, Germany). Reference standards including chlorogenic acid, caffeic acid, rosmarinic acid, and salicylic acid, as well as gallic acid, catechin, rutin, and quercetin, were obtained from Sigma-Aldrich (USA).

For the biological assays, human A549 (lung epithelial) and AGS (gastric adenocarcinoma) cell lines, along with Normal Human Dermal Fibroblasts (NHDF), were obtained from the Iranian Biological Resource Center (IBRC; Tehran, Iran). Cell culture media (DMEM, RPMI-1640, and PCS-201-030), Fetal Bovine Serum (FBS), penicillin/streptomycin, and L-glutamine were purchased from Gibco (Thermo Fisher Scientific, USA). 3-[4,5-dimethylthiazol-2-yl]-2,5-diphenyltetrazolium bromide (MTT) and 4,6-diamidino-2-phenylindole (DAPI) were sourced from Sigma-Aldrich (USA). For molecular analysis, the RiboEX total RNA extraction kit (Cat. No. 302-001) was obtained from GeneAll (Seoul, Korea). The first-strand cDNA synthesis kit (Cat. No. A101161) and PowerUp SYBR Green Master Mix were purchased from Pars Tous Biotechnology (Mashhad, Iran).

### Plant collection and extraction

*Nepeta glomerulosa* specimens were collected at an elevation of 2,203 m in the Binalod Mountains of Khorasan Razavi Province (latitude 36.16.826 N, longitude 59.07.484 E). The voucher specimen (FUMH 36809) was deposited in FUMH and identified by M.R. Joharchi.

The plant material was air-dried in the shade under well-ventilated conditions until reaching a constant weight. The hydroalcoholic extract was prepared by maceration. Thirty grams of the dried twig powder were placed in a glass jar with 500 mL of 80% ethanol and incubated in a shaker incubator (Innova 42, New Brunswick Scientific) at 40 °C and 100 rpm for 24 h. All solvents were analytical-reagent grade (Merck Chemical Inc., Darmstadt, Germany). After extraction, the mixture was filtered through Whatman no. 1 filter paper, and the filtrate was collected. Concentration under reduced pressure (40 °C, 150–180 mbar, and 40 rpm) using a rotary evaporator (Heidolph WB eco, Germany) produced the crude hydroalcoholic extract [19]. To support reproducibility, extract batches were standardized using the HPLC marker profile described below.

### HPLC evaluation of the extract

A total of 0.1 g of dried extract sample was dissolved in 2 mL of methanol, followed by the addition of 2 mL of phosphate buffer. The solution was filtered through a 0.45 μm membrane filter and injected into an HPLC system (RIFR-WI-PHYT-29) equipped with a UV detector. The HPLC analysis was performed using an Agilent 1260 rapid technology system with a Ultisil XB-C18 column (5 μm, 250 mm × 4.6 mm i.d). Column conditioning was carried out using methanol as the first mobile phase and phosphate buffer (pH 2.3) as the second. Gradient elusion was performed at 30 °C with a flow rate of 1 mL/min. Detection was conducted at wavelengths of 254, 280, 300, and 330 nm using a PDA detector. The concentrations of chlorogenic acid, caffeic acid, Rosmarinic acid, and Salicylic acid were quantified based on calibration curves and expressed relative to the weight of the dried extract [20]. Reference standards of gallic acid, catechin, rutin, and quercetin were included in the analysis, but none of these compounds were identified in the examined samples. HPLC-UV/PDA was used for targeted quantification of defined marker phenolic acids for batch standardization; comprehensive LC-MS profiling was outside the scope of this study and is suggested for future work.

### Preparation of nanoemulsion by the extract

A vitamin D₃–fortified MCT (Medium Chain Triglycerides) oil containing the plant extract (5–10% w/v; oil phase, 30% v/v) was combined with an aqueous phase (70% v/v) composed of pre-formed, stabilized silver nanoparticles (20–50 nm, dispersed in deionized water). Tween 80 (3–5% w/w) was incorporated as the non-ionic emulsifier in the aqueous phase before emulsification. The oil phase was added dropwise to the aqueous phase under mechanical stirring (1,000–2,000 rpm) to obtain a coarse emulsion. The pre-emulsion was then processed by high-pressure homogenization (≈ 50–140 MPa; 10,000–20,000 psi; 3–5 passes) followed by pulsed probe sonication (20 kHz, 200–400 W, 2–4 min total) with intermittent cooling/ice-bath to limit temperature rise. All steps were conducted under temperature control (≤ 25 °C) and protected from light to minimize vitamin D₃ degradation and preserve AgNP stability. No silver salts or reduce agents were used at any stage. The nanoemulsion was stored protected from light until characterization [21, 22, 23].

The physical stability of the synthesized Ag-N.g.Ext. NEs was monitored through visual observation for any phase separation, creaming, or precipitation. The nanoemulsion remained stable and homogenous at 4 °C for at least six months. This long-term stability is attributed to the high electrostatic repulsion between droplets (confirmed by a high zeta potential of (-23.1 mV) and the steric stabilization provided by the Tween 80 surfactant, which prevents droplet coalescence and Ostwald ripening [7, 8].

### Characterization of nanoemulsion

The physicochemical properties of the synthesized nanoemulsion were characterized by employing complementary techniques. Particle size distribution and polydispersity index (PDI) were analyzed by dynamic light scattering (DLS) on a Malvern Mastersizer X (Malvern, UK) after diluting samples 1:100 in deionized water to minimize multiple scattering [24].

Zeta potential was determined by Laser Doppler Electrophoresis using a Malvern Zetasizer (UK), with samples diluted 1:1,000 in Milli-Q water to ensure conductivity and reduce aggregation [24].

Morphological features were observed by scanning electron microscopy (SEM, Zess DMS960-A). Dried droplets of the nanoemulsion were sputter-coated with a thin gold layer, and images were captured under a 15 kV accelerating voltage [25].

For chemical characterization, Fourier-transform infrared (FTIR) spectra were recorded on a JASCO FTIR-4600 spectrometer across 400-4,000 cm^− 1^ to identify functional groups and confirm chemical integrity [26].

Structural analysis was carried out using X-ray diffraction (XRD, Philips PW1730) over a 2θ range of 0.998 °- 99.95 ° with a 0.026° step size. The crystalline nature of the silver nanoparticles was verified, and crystalline sizes were calculated using the Scherrer equation [27]. XRD was recorded on dried residues of the nanoemulsion to verify the crystalline phase of the silver-containing fraction.

### Cell culture

A549 cells were maintained in DMEM, AGS in RPMI-1640, and HDF in PCS-201-030 fibroblast medium. All media were supplemented with 10% FBS, 100 U/mL penicillin, 100 µg/mL streptomycin, and 2 mM L-glutamine (Gibco). Cultures were incubated at 37 °C, 5% CO₂, and subcultured every 3–4 days.

### MTT cytotoxicity assay

Cytotoxicity of *N. glomerulosa* extract, silver nanoparticles (AgNPs), and *N. glomerulosa* extract-loaded nanoemulsion (Ag-N.g.Ext. NEs) was assessed on A549, AGS, and NHDF via MTT. Cells were seeded at 1 × 10⁴ cells/well in 96-well plates and incubated for 24 h. Treatments (extract: 0–1500 µg/mL; AgNPs and Ag-N.g.Ext. NEs: 0–500 µg/mL) were added (*n* = 3), and viability was measured at 24, 48, and 72 h. At each well, 20 µL of 5 mg/mL MTT was added per well for 4 h, the medium was removed, and the formazan crystals were dissolved in 50 µL DMSO (wrapped in foil) for 1 h. Absorbance at 570 nm was read on an ELISA reader (Milton Roy- Spectronic21D- USA) [28, 29]. Cell viability was calculated based on the following formula. To reduce potential optical interference from dispersed nanoparticles, the culture medium containing the formulations was removed before dissolving formazan crystals and reading absorbance.

$$Viability\;(\%)=\left(\frac{sample\;A_{570}}{control\;A_{570}}\right)\times100$$

### Apoptosis rate in DAPI assay

To assess apoptosis by DAPI staining (4,6-diamidino-2-phenylindole**)**, A549 and AGS cells were seeded in 6-well plates and treated with plant extract at 500 µg/mL and 250 µg/mL, respectively. In parallel, both lines were exposed to 125 µg/mL of Ag-N.g.Ext. NEs (their IC₅₀). After treatment, the medium was removed, cells were washed with PBS, fixed in 4% paraformaldehyde, and stained with 300 nM DAPI for 5 min in the dark. Nuclear morphology was then examined by fluorescence microscopy. An Olympus IX81 inverted fluorescence microscope equipped with an Olympus DP70 camera (Olympus, Japan) [30].

### Gene expression by Real-time PCR

A549 and AGS cells were seeded in 6-well plates and treated for 24 h with their respective IC₅₀ concentrations of *N. glomerulosa* extract (500 µg/mL for A549; 250 µg/mL for AGS) or with the Ag-N.g.Ext. NEs (125 µg/mL for both cell lines). Total RNA was extracted from 5 × 10⁶ cells using the RiboEX kit (GeneAll, Korea) and quantified with a NanoDrop spectrophotometer (Srcituro BiiketoiB). First-strand cDNA was synthesized from 1 µg RNA using the Pars Tous cDNA Synthesis Kit (Iran; Cat. A101161) following the manufacturer’s protocol, and subsequently treated with RNase H (1 µL, 20 min at 37 °C). The resulting cDNA was either stored at − 20 °C. Quantitative real-time PCR was performed on a Rotor-Gene 6000 system (QIAGEN) in 25 µL reactions containing 12.5 µL of the PowerUp™ SYBR_ Green Master Mix (Pars Tous Biotechnology, Iran), 1 µL cDNA, 1 µL of each primer (0.2 pmol/µL), and 9.5 µL nuclease-free water. Primers were designed using the Primer3 (ver.0.4.0) tool and Gen Runner (ver.6) software. Primer sequences were as follows:

TP53 (93 bp): F 5′-GCGAGCACTGCCCAACAACAC-3′; R 5′-TCACGCCCACGGATCTGAAGG-3′.

BAX (133 bp): F 5′-TGCCTCAGGATGCGTCCACC-3′; R 5′-CCCCAGTTGAAGTTGCCGTC-3′.

GAPDH (122 bp): F 5′-CTCATTTCCTGGTATGACAACG-3′; R 5′-CTTCCTCTTGTGCTCTTGCT-3′.

Thermal cycling conditions were initial denaturation at 95 °C for 4 min, followed by 35 cycles of 94 °C for 30 s, 60 °C for 30 s, and 72 °C for 5 s, with a final extension at 72 °C for 5 min. A melt-curve analysis was performed from 70 to 95 °C to verify amplification specificity. All reactions were carried out in triplicate, and relative gene expression was calculated using REST 2009 software. Gene expression was normalized to GAPDH as a housekeeping gene. After obtaining E, the following formula was used to calculate the fold change of each gene in Excel 2016 [28, 29].

$$Fold\;change=\frac{\left(Et\right)^{\triangle Ct\; target\;(control-sample)}}{\left(Eref\right)^{\triangle Ct\;ref(control-sample)}}$$

### Statistical analysis

Half-maximal inhibitory concentration (IC₅₀) values were calculated from the normalized averages of three independent MTT assays and compared across different cell lines using two-way analysis of variance (ANOVA), followed by Tukey’s post hoc test. Relative gene expression was quantified by the ΔΔCt method, assuming 100% PCR efficiency, with three technical replicates analyzed in REST2009. The resulting ΔCt values were subjected to two-way ANOVA. All statistical analyses were conducted using GraphPad Prism (ver.10) and SPSS (ver.21), with a significance threshold set at *P* < 0.05.

## Results

### HPLC analysis of the crude extract

**Fig. 1:**
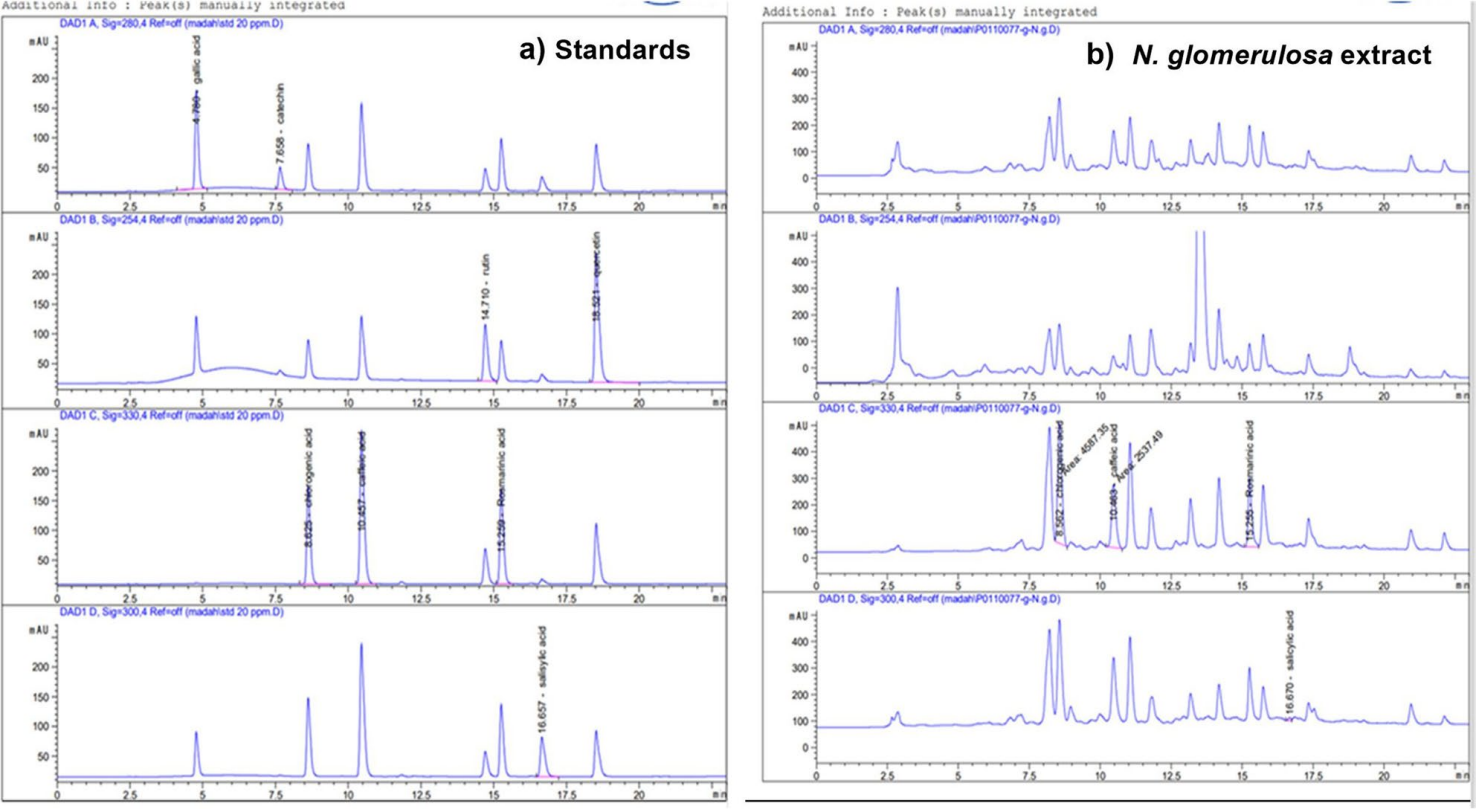
HPLC chromatograms (**a**) standards, (**b**) chromatograms of the *N. glomerulosa* species showing the presence of four phenolic acids

Analysis of the *N. glomerulosa* extract revealed the absence of Gallic acid, Catechin, Rutin, and Quercetin. Instead, four phenolic acids, i.e., Chlorogenic acid, Caffeic acid, Rosmarinic acid, and Salicylic acid, were detected, among which chlorogenic acid was the most abundant, exhibiting the highest concentration (Fig. 1). The concentration of each phenolic acid is summarized in Table 1.

**Table 1:** Quantitative analysis of four phenolic acids i.e., Chlorogenic acid, Caffeic acid, Rosmarinic acid, and Salicylic acid in the *N. glomerulosa* species extract, determined by HPLC (mg/g dry weight)

Phenolic acids	Chlorogenic acid	Caffeic acid	Rosmarinic acid	Salicylic acid
Contents (mg/g)	2.69	0.86	1.41	0.1

### Characterization of silver nanoparticles and nanoemulsion

**Fig. 2:**
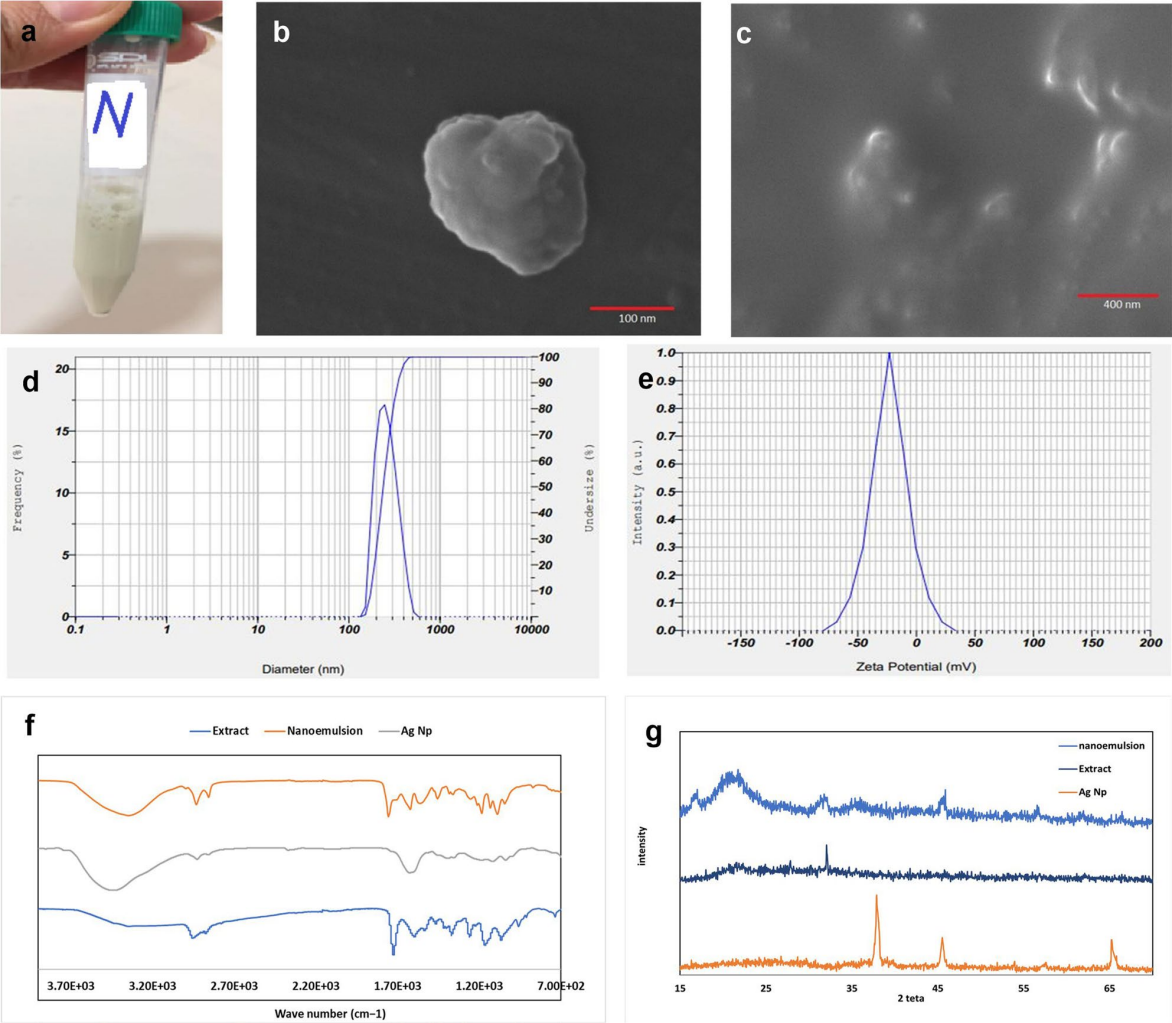
Characterization of Ag-N.g.Ext. NEs by different techniques: **a **The synthesized nanoemulsion; **b-c **Morphology by SEM at two different magnifications; **d** Particle size distribution diagram by DLS; **e** Zeta potential diagram by Laser Doppler Electrophoresis, **f** FTIR, **g** XRD

The synthesized Silver-Loaded Nanoemulsion of *Nepeta glomerulosa* Extract (Ag-N.g.Ext. NEs) appeared as a stable, milky/opalescent homogeneous dispersion (Fig. 2a), characteristic of nanometric droplets. Scanning electron microscopy (SEM) images (Fig. 2b, c) revealed well-defined, spherical morphology with a uniform distribution. The absence of significant agglomeration suggests that the bioactive compounds from the plant extract successfully acted as capping agents, stabilizing the silver core within the nanoemulsion droplets.

Dynamic light scattering (DLS) analysis showed an average hydrodynamic diameter of 269.5 nm (Fig. 2d), confirming the formation of a stable nanoemulsion. The polydispersity index (PDI) indicated a narrow size distribution. Furthermore, the zeta potential was measured at a significant negative value (Fig. 2e), indicating strong electrostatic repulsion between the droplets, which ensures the long-term physical stability of the formulation.

The chemical interactions were investigated using FTIR spectroscopy (Fig. 2f). The broad peak at 3349 cm⁻¹ (O–H stretching) and the prominent peaks in 1744 and 1641 cm⁻¹ (C = O stretching) are attributed to the polyphenols and proteins present in the plant extract. The noticeable shifts in these bands in the Ag-N.g.Ext. NEs compared to the raw extract confirm that these functional groups were actively involved in the dual role of reduction (Ag⁺ to Ag⁰) and capping of the silver nanoparticles.

The crystalline structure was further validated by XRD analysis (Fig. 2g). The diffraction pattern of the silver nanoparticles (AgNPs) exhibited sharp characteristic peaks at 2theta values of approximately 38.1°, 44.3°, and 64.5°, corresponding to the (111), (200), and (220) planes of the face-centered cubic (FCC) silver crystals. In the nanoemulsion (Ag-N.g.Ext. Nes) profile (blue curve), these peaks are still detectable alongside a broad amorphous halo at 20° (originating from the oil phase and extract components). This confirms the successful incorporation of crystalline silver nanoparticles into the nanoemulsion matrix. The observed peak broadening is consistent with the nanoscale dimensions of the silver crystallites, as predicted by the Scherrer equation.

### Cytotoxicity evaluation

**Fig. 3:**
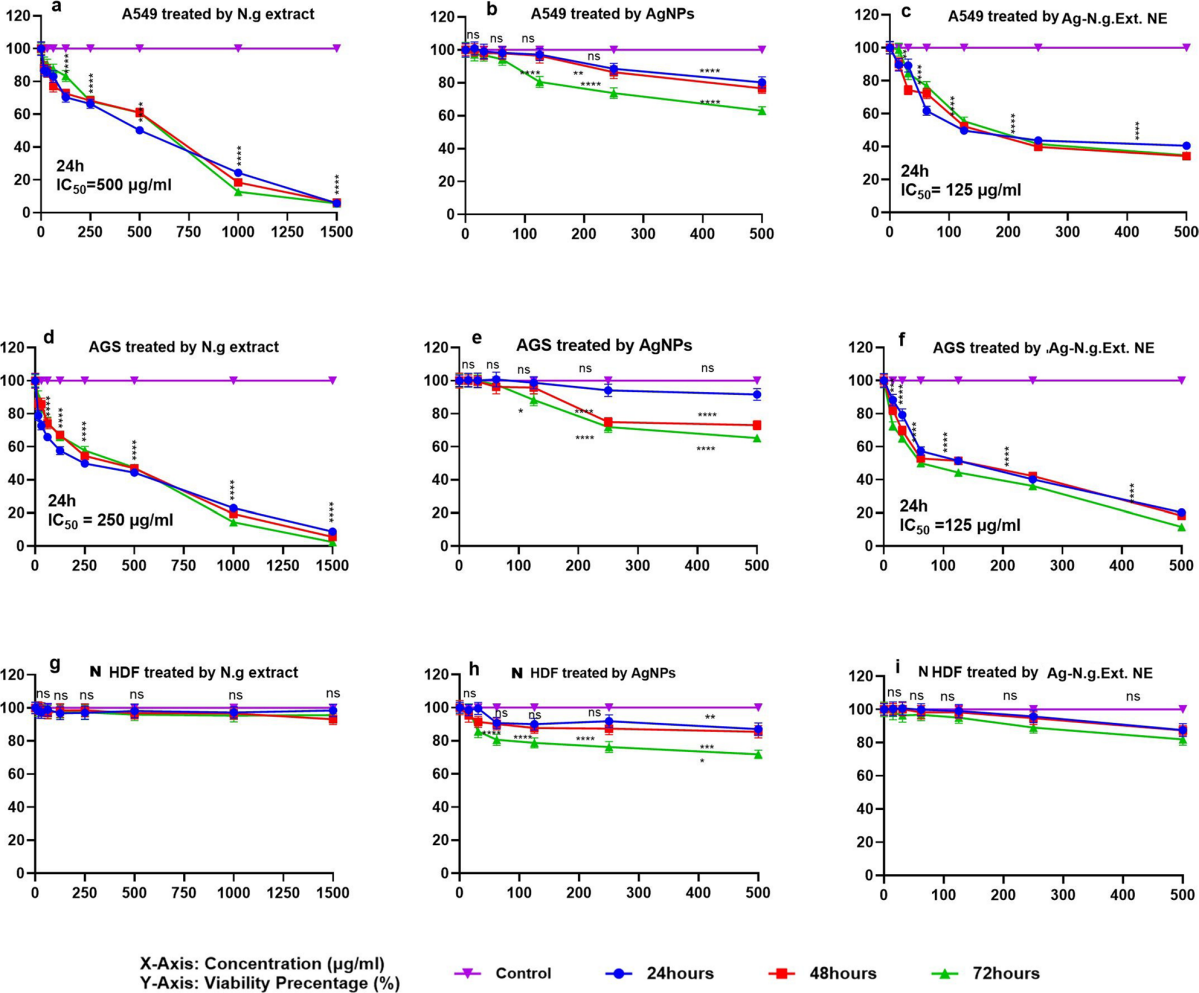
The cell viability of the A549 lung and AGS gastric cancer cell lines as well as NHDF normal cells, during 24, 48, and 72 h post-inoculation time under different treatments: A549 cancer cells treated with (**a**) *N. glomerulosa* crude extract; (**b**) silver nanoparticles, and (**c**) Silver-loaded nanoemulsion of *N.glomerulosa* extract respectively. AGS cancer cells treated with (**d**) *N. glomerulosa* crude extract; (**e**) silver nanoparticles, and (**f**) Silver-loaded nanoemulsion of *N.glomerulosa* extract respectively. NHDF cells treated with (**g**) *N. glomerulosa* crude extract; (**h**) silver nanoparticles, and (**i**) Silver-loaded nanoemulsion of *N.glomerulosa* extract respectively. *N.g extract*: extract of *Nepeta glomerulosa;Ag.NPs*: silver nanoparticles; *Ag-N.g.Ext. NEs*: Silver-loaded nanoemulsion of *N.glomerulosa* extract. (*significant difference at *P* < 0.05; **significant difference at *P* < 0.01; *** significant difference at *P* < 0.001; **** significant difference at *P* < 0.0001; ns: not significant)

The cytotoxic effects of *N*. *glomerulosa* extract, AgNPs, and the Ag-N.g.Ext. NEs were assessed by the MTT assay (570 nm) in A549, AGS, and NHDF cells at 24, 48, and 72 h, respectively. As illustrated in Fig. 3, all treatments elicit a dose-dependent decline in cell viability, with the strongest effect observed at 24 h. The crude extract exhibited IC₅₀ values of 500 µg/mL for A549 (Fig. 3a) and 250 µg/mL for AGS (Fig. 3d), while AgNPs displayed only limited cytotoxicity separately. In contrast, incorporating the extract into Ag-N.g.Ext. NEs significantly enhanced its efficacy, producing an IC₅₀ of Approximately 125 µg/mL in both cancer cell lines (Fig. 3c, f). The determined IC₅₀ values were subsequently used for apoptosis assays and gene-expression analyses. Notably, none of the treatments compromised the viability of Normal Human dermal fibroblast (NHDF) under the tested conditions, suggesting preferential cytotoxicity toward malignant cells (Fig. 3g-i).

### DAPI assay

**Fig. 4:**
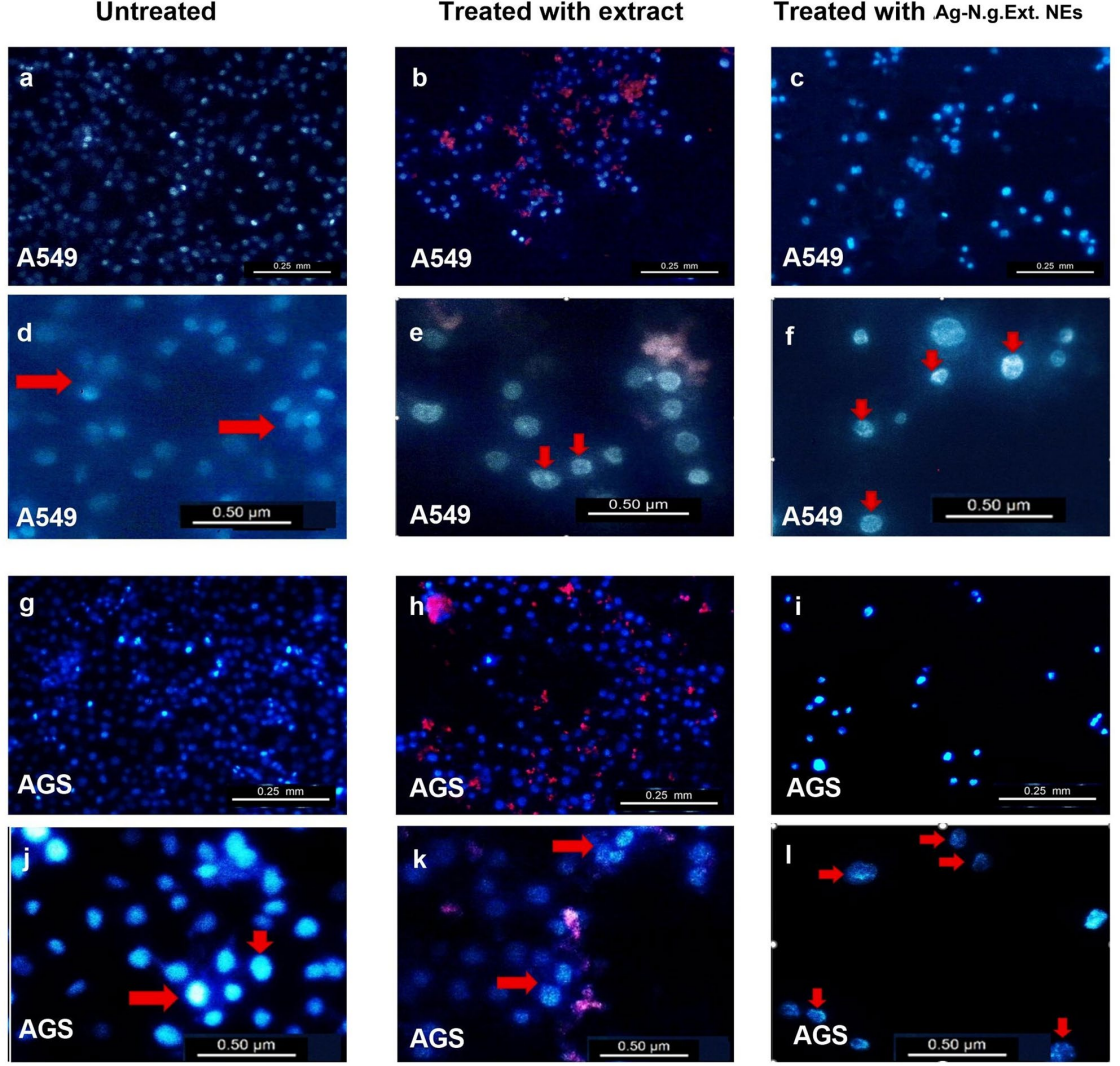
Fluorescence microscopy images of A549 (**a-f**) and AGS (**g-i**) cells with two magnifications and under different treatment groups, i.e., untreated, treated with extract, and treated with silver-loaded nanoemulsion of *N.glomerulosa* extract (Ag-N.g.Ext. NEs ). The nuclei appeared blue under DAPI staining. In cells treated with the extract (**b**,** h**) or silver-loaded nanoemulsion of *N.glomerulosa* extract (**c**,** i**), the cell number decreased, and a subset of nuclei (red arrow) showed irregular, punctuated DAPI staining, indicative of apoptosis (**e**,** f**,** k**,** l**), unlike untreated controls (**d**,** j**)

DAPI-stained images of AGS and A549 cells after 24 h are shown in Fig. 4. Two magnifications are applied for each treatment group. The untreated control cells display uniformly shaped, intensely stained blue nuclei, reflecting healthy chromatin structure (Fig. 4d, j).

In samples treated with the extract and Silver-loaded nanoemulsion of *N.glomerulosa* extract (Ag-N.g.Ext. NEs) at the IC_50_ concentration, overall cell density was reduced (Fig. 4b, c, h, i). The reduction in cell count was more pronounced in the nanoemulsion-treated.

Moreover, many nuclei, especially in the nanoemulsion group, appeared irregular, fragmented, and dotted, which are classic indicators of apoptosis (Fig. 4f, l).

### *TP53* and *BAX* gene expression in AGS and A549 cells

**Fig. 5:**
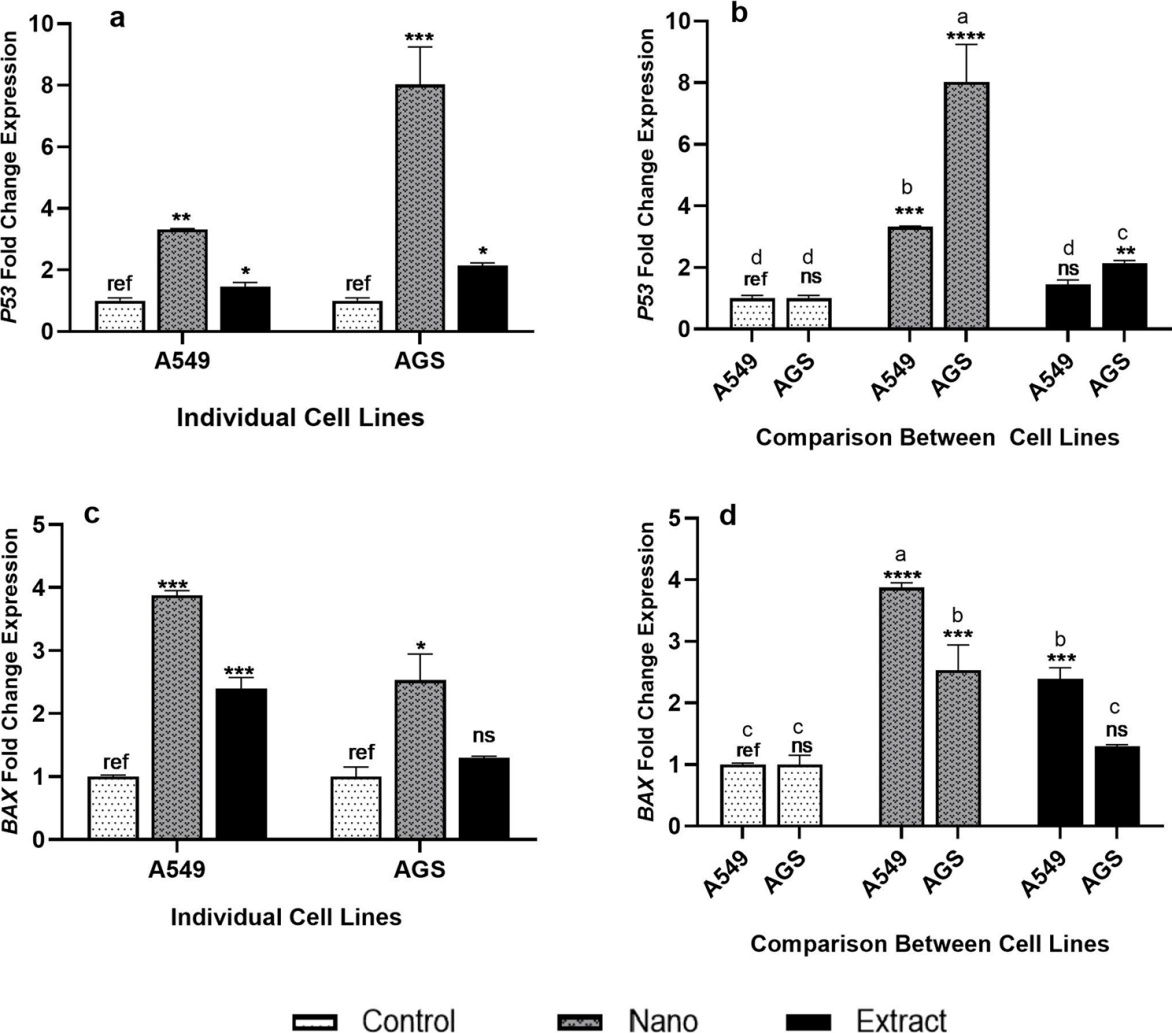
The effects of *N. glomerulosa* extract and its silver-loaded nanoemulsion of *N.glomerulosa* extract on apoptosis-related gene expression in gastric cancer (AGS) and lung cancer (A549) cell lines: (**a-b**) Relative fold change expression in *Tp53* gene expression for each cell line separately (**a**) and comparison between cell lines (**b**). (**c-d**) Relative fold change expression in the *BAX* gene expression for individual cell lines (**c**) and comparison between cell lines (**d**). Data are presented as mean ± SD (*n* = 3). Significant differences are indicated by letters and asterisks. (*significant difference at *P* < 0.05; **significant difference at *P* < 0.01; *** significant difference at *P* < 0.001; **** significant difference at *P* < 0.0001; ns: nonsignificant; ref: reference group)

Relative to untreated controls, *TP53* expression increased following treatment with the crude extract in both cell lines (A549: 1.45-fold; AGS: 2.14-fold; *P* < 0.05; Fig. 5a). Encapsulation of the extract in the silver nanoemulsion further augmented *TP53* expression (A549: 3.32-fold, *P* < 0.01; AGS: 8.03-fold, *P* < 0.001; Fig. 5a). For *BAX*, A549 cells showed significant induction with both formulations (2.39-fold with the crude extract; 3.88-fold with the nanoemulsion (Ag-N.g.Ext. NEs), *P* < 0.001; Fig. 5c), whereas in AGS cells only the nanoemulsion (Ag-N.g.Ext. NEs) produced a significant increase (2.53-fold, *P* < 0.05); the 1.30-fold rise with the crude extract was not significant (Fig. 5c). Cross-comparison (Fig. 5b, d) indicated that nanoemulsion-mediated upregulation of *TP53* was greater in AGS than in A549, while *BAX* induction was more pronounced in A549.

## Discussion

The present study investigated the anticancer activity of *Nepeta glomerulosa* hydroalcoholic crude extract and its silver-loaded nanoemulsion of *N.glomerulosa* extract in A549 and AGS cancer cell lines and evaluated apoptosis-related outcomes including *TP53* and *BAX* expression. The nanoemulsion enhanced cytotoxicity and apoptosis compared with the crude extract, as shown by lower IC₅₀ values, increased nuclear fragmentation in DAPI assays, and upregulation of *TP53* and *BAX* expression.

The phytochemical profile of *N. glomerulosa* extract revealed four main polyphenols, i.e., Chlorogenic acid, Caffeic acid, Rosmarinic acid, and Salicylic acid, which have been reported to exhibit antioxidants, anti-inflammatory, and anticancer activities. Rosmarinic acid can suppress metastasis by inhibiting migration, inducing cell cycle arrest, and enhancing apoptosis signaling, thereby reducing tumorigenicity in various cancers [31]. Chlorogenic acid also exhibits anticancer potential by inducing cell cycle arrest, promoting apoptosis, and inhibiting malignant cell proliferation [32, 33]. Caffeic acid triggers apoptosis through ROS elevation and mitochondrial disruption [34]. Caffeic acid phenethyl ester (CAPE) further suppresses cell growth, increases nuclear fragmentation, and activates the BAX protein, leading to mitochondrial apoptosis [35, 36]. Salicylic acid contributes to anticancer activity through anti-inflammatory and apoptotic mechanisms [35]. In this study, chlorogenic acid (2.69 mg/g) and rosmarinic acid (1.41 mg/g), the most abundant phenolic constituents in the extract, may contribute to the observed cytotoxic activity of both the crude extract and its nanoemulsion; however, due to the multicomponent nature of the extract, activity cannot be attributed to a single constituent. The high diversity and rich content of terpenoids, flavonoids, and phenolic compounds in *Nepeta* species contribute to their pharmacological activities. Fan et al. (2017) demonstrated the anticancer properties of total flavonoids extracted from *Nepeta cataria* L. against the A549 lung cancer cell line [37]. Similarly, Köngül Şafak et al. (2022) reported that the cytotoxic properties of several *Nepeta* species against breast cancer cell lines were mainly associated with the presence of saponins and terpenoids [38]. The chloroform extract of *Nepeta deflersiana* Schweinf. Ex-Hedge was shown to reduce the viability of MCF-7 cells, with A549 cells exhibiting greater sensitivity [39], whereas in the present study, AGS gastric cancer cells displayed higher sensitivity to the hydroalcoholic extract of *N. glomerulosa* compared to A549 lung cancer.

Although polyphenols hold therapeutic potential, their poor solubility, rapid metabolism, and low bioavailability limit their efficacy. Incorporating *N. glomerulosa* extract into a silver-loaded nanoemulsion enhanced its potency, as evidenced by a reduced IC₅₀. The significant reduction in IC50 values upon nanoemulsification (from 500 to 250 µg/mL in extract treated to 125 µg/mL) can be attributed to the Enhanced Permeability and Retention (EPR)-like effect at the cellular level [40]. The nanoemulsion droplets act as ‘Trojan horses,’ protecting the sensitive polyphenols from premature degradation and facilitating their intracellular release [41]. Furthermore, the presence of silver nanoparticles within the droplets creates a synergistic oxidative stress (ROS generation), which effectively lowers the concentration of extract required to induce apoptosis [42]. The observed selectivity, demonstrated by the lack of toxicity towards normal dermal fibroblasts (NHDF), highlights the potential of this formulation as a targeted delivery system that exploits the higher metabolic activity and endocytic rate of cancer cells compared to normal cells [40, 42].

Nanoemulsions, characterized by nanoscale droplet sizes and high surface-to-volume ratios, offer superior physical stability and enhanced bioavailability, making them ideal carriers for bioactive compounds and metallic nanoparticles [7, 8, 43]. In this study, the average hydrodynamic diameter was found to be 269.5 nm. This size range (20–500 nm) is optimal for bypassing rapid splenic filtration and enhancing cellular uptake through endocytosis [7, 8, 44]. The polydispersity index (PDI) and the single peak observed in DLS analysis (Fig. 2d) indicate a narrow size distribution, which is crucial for the long-term kinetic stability of the formulation. The negative zeta potential observed (Fig. 2e) is a key indicator of physical stability. This surface charge is likely attributed to the adsorption of anionic phytochemicals from the plant extract (such as phenolics and organic acids) onto the AgNPs and the oil-water interface [42, 44]. This negative cloud creates a strong electrostatic repulsion, preventing droplet coalescence and nanoparticle agglomeration that facilitate interaction with cancer cell membranes [6, 7, 42, 44].

FTIR analysis confirmed the successful ‘Green Synthesis’ and capping process. The shift in functional groups, particularly aldehydes, ketones, and carboxylic acids, suggests that these biomolecules acted as reducing agents for Ag^+^ ions and subsequently anchored to the silver surface as stabilizing ligands. This capping mechanism is vital for maintaining the nanometric dimensions of silver particles within the emulsion matrix [42, 44].

Furthermore, the XRD pattern (Fig. 2g) provided definitive evidence of the crystalline nature of the silver phase. The distinct diffraction peaks observed are characteristic of the face-centered cubic (FCC) structure of metallic silver. While the broad halo at lower angles reflects the amorphous nature of the nanoemulsion’s organic phase (oil and extract), the sharp peaks at 38°, 44°, and 65° confirm that the silver nanoparticles maintained their high crystallinity even after being encapsulated within the nanoemulsion droplets. This hybrid system (Ag-N.g.Ext. NEs) combines the potent antimicrobial/anticancer properties of silver with the superior solubility and penetrability of nanoemulsions [7, 8, 42].

Silver nanoparticles are known to generate reactive oxygen species (ROS), leading to DNA damage and TP53 stabilization, thereby altering apoptosis-related gene expression [45, 46, 47]. In this study, loading *Nepeta glomerulosa* extract into a silver-based nanoemulsion enhanced pro-apoptotic activity in A549 and AGS cells, as reflected by lower IC₅₀ values, nuclear fragmentation, and upregulation of TP53 and BAX compared with the crude extract. The stronger BAX response in A549 and marked Tp53 upregulation in AGS suggest engagement of the p53-BAX axis, promoting mitochondrial permeabilization and apoptosis [47, 48, 49].

In parallel, silver nanomaterials are well known to induce oxidative DNA damage in epithelial cancers, including A549, by stabilizing p53 and driving BAX-dependent mitochondrial apoptosis [49]. This may explain the superior activity of the extract-loaded nanoemulsion compared to the free extract in our system [50]. Similar findings were reported by Thuy et al. (2023), who showed that nanoemulsion composed of α-tocopherol succinate and dequalinium exhibited mitochondria-targeting and anticancer effects [51]. Likewise, Yu et al. (2017) demonstrated that caffeic acid phenethyl ester induced apoptosis in oral cancer cells via BAX and Puma upregulation [35].

Previous studies have focused on the anticancer effects of other *Nepeta* species. For example, evaluation of *Nepeta nuda* extract in cancer cell lines, including MDA-MB-231 and MCF7 (breast), HT29 and Colon 26 (colon), HepG2 (liver), as well as on a non-cancerous skin cell line, revealed significant inhibition of colony formation in colon cancer cells. It also exhibited marked pro-apoptotic activity involving *CASP8* and increased the expression of *ATG3* and *BECN1*, indicating that autophagic cell death contributes to its antitumor effects [17]. Daei et al. (2022) showed that silver nanoparticles induce apoptosis in bladder cancer cells through ROS generation, upregulation of the Bax/Bcl-2 ratio, and activation of caspases-3 and 7 [52]. Comparative studies with standard chemotherapeutics, combination regimens, and in vivo tumor models will be the next crucial steps to advance this approach toward clinical application.

Limitations: Toxicity evaluation was limited to an in vitro HDF model, and nanoparticle-related interference with colorimetric assays (including MTT) cannot be fully excluded. In addition, mechanistic evidence is limited to apoptosis-related morphology (DAPI/AO-EB) and TP53/BAX expression, and broader pathway confirmation is warranted. Future work should include orthogonal viability assays and in vivo biodistribution/safety assessment of silver-containing formulations [53, 54].

## Conclusion

The silver-loaded nanoemulsion of *N. glomerulosa* extract demonstrated improved in vitro anticancer activity compared with the crude extract and was associated with apoptosis-related changes, including nuclear fragmentation and increased TP53/BAX expression in lung and gastric cancer cells. By harnessing the bioactivity of phytochemicals and the delivery advantages of nanotechnology, this strategy may help address limitations of natural compounds such as poor solubility and low bioavailability. Further, in vivo studies are warranted to validate safety, biodistribution, and anticancer efficacy.

## Data Availability

Availability of data and materialThe datasets generated during and/or analysed during the current study are not publicly available but are available from the corresponding author on reasonable request.

## Abbreviations

A549: Adenocarcinoma Human Alveolar Basal Epithelial Cells
AGS: Adenocarcinoma Gastric Cells
BAX: The B-cell lymphoma 2 (Bcl-2)-associated X protein
CAPE: Caffeic acid phenethyl ester
DAPI: 4,6-diamidino-2-phenylindole
DLS: Dynamic Light Scattering
DMEM: Dulbecco’s Modified Eagle’s Medium
DMSO: Dimethyl sulfoxide
FBS: Fetal bovine serum
FTIR: Fourier Transform Infrared Spectroscopy
GAPDH: Glyceraldehyde 3-phosphate dehydrogenase
HDF: Normal dermal fibroblasts
HPLC: High Performance Liquid Chromatography
IC_50_: half-maximal inhibitory concentrations
MCT: Medium Chain Triglycerides
SEM: Scanning Electron Microscopy
TP53 (p53): The tumor protein P53
RPMI: Roswell Park Memorial Institute medium
XDR: X-ray Diffraction
